# Fracture Behavior of a 2D Imine‐Based Polymer

**DOI:** 10.1002/advs.202407017

**Published:** 2024-09-12

**Authors:** Bowen Zhang, Xiaohui Liu, David Bodesheim, Wei Li, André Clausner, Jinxin Liu, Birgit Jost, Arezoo Dianat, Renhao Dong, Xinliang Feng, Gianaurelio Cuniberti, Zhongquan Liao, Ehrenfried Zschech

**Affiliations:** ^1^ Fraunhofer Institute for Ceramic Technologies and System (IKTS) Maria‐Reiche‐Straße 2 01109 Dresden Germany; ^2^ Faculty of Electrical and Computer Engineering Technical University of Dresden 01062 Dresden Germany; ^3^ Faculty of Chemistry and Food Chemistry Technical University of Dresden 01062 Dresden Germany; ^4^ Institute for Materials Science and Max Bergmann Center for Biomaterials Technical University of Dresden 01062 Dresden Germany; ^5^ College of Chemistry and Chemical Engineering Lanzhou University Lanzhou 730000 P. R. China; ^6^ Key Laboratory of Colloid and Interface Chemistry of the Ministry of Education School of Chemistry and Chemical Engineering Shandong University Jinan 250100 P. R. China; ^7^ Dresden Center for Computational Materials Science (DCMS) Technical University of Dresden 01062 Dresden Germany

**Keywords:** 2D polymer, fracture mechanisms, in situ test, transmission electron microscope (TEM)

## Abstract

2D polymers have emerged as a highly promising category of nanomaterials, owing to their exceptional properties. However, the understanding of their fracture behavior and failure mechanisms remains still limited, posing challenges to their durability in practical applications. This work presents an in‐depth study of the fracture kinetics of a 2D polyimine film, utilizing in situ tensile testing within a transmission electron microscope (TEM). Employing meticulously optimized transferring and patterning techniques, an elastic strain of ≈6.5% is achieved, corresponding to an elastic modulus of (8.6 ± 2.5) GPa of polycrystalline 2D polyimine thin films. In step‐by‐step fractures, multiple cracking events uncover the initiation and development of side crack near the main crack tip which toughens the 2D film. Simultaneously captured strain evolution through digital image correlation (DIC) analysis and observation on the crack edge confirm the occurrence of transgranular fracture patterns apart from intergranular fracture. A preferred cleavage orientation in transgranular fracture is attributed to the difference in directional flexibility along distinct orientations, which is substantiated by density functional‐based tight binding (DFTB) calculations. These findings construct a comprehensive understanding of intrinsic mechanical properties and fracture behavior of an imine‐linked polymer and provide insights and implications for the rational design of 2D polymers.

## Graphical abstract

This work performs an in situ tensile testing within a transmission electron microscope to quantitatively study the fracture kinetics of a 2D polyimine film. Apart from quantifying mechanical properties, the crack interactions and occurrence of transgranular fracture with directional mechanical flexibility are uncovered. This work provides fundamental understanding about the fracture behavior of 2D polymers and illuminates structural designs for applications.

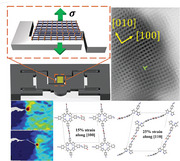

## 1 Introduction

2D materials, such as graphene, graphene oxide (GO), boron nitride and transition metal dichalcogenides (TMDs) have been widely studied since being discovered because of their outstanding physical properties.^[^ ^1^, ^2^, ^3^ ^]^ Many studies explore the mechanical properties of 2D materials by mechanical testing using atomic force microscopy (AFM) and nanoindentation.^[^ ^4^, ^5^, ^6^ ^]^ However, since the fracture mechanisms on the nanoscale are different from those on the macroscale, size‐dependent properties, e.g., the fracture toughness in 2D material systems must be determined and investigated in the nanoscale. This applies, e.g., for the kinetics of microcrack evolution. As the benchmark 2D material, graphene initiates these studies, and its fracture mechanism has been extensively and quantitatively verified.^[^ ^7^, ^8^, ^9^, ^10^ ^]^ Afterward, multilayer graphene,^[^ ^11^, ^12^ ^]^ GO,^[^ ^13^ ^]^ MoSe_2_,^[^ ^14^, ^15^ ^]^ MoS_2_,^[^ ^15^, ^16^ ^]^ and hexagonal boron nitride (h‐BN)^[^ ^17^ ^]^ have been subsequently studied. Because of the brittle nature of 2D materials and the perspective to use them in, e.g., flexible electronic devices, it must be studied how their mechanical properties can be optimized for such applications.

Recently, highly crystalline 2D polymers, particularly covalent organic frameworks (COFs), which is a special class of 2D materials, constructed by periodic covalent linkage of various organic building blocks in orthogonal direction^[^ ^18^, ^19^ ^]^ have emerged and attracted a great deal of interest in broad flexible applications including electronics/optoelectronics, energy storage/conversion, sensing, gas separation, and catalysis.^[^ ^20^, ^21^, ^22^, ^23^, ^24^ ^]^ The possible chemical and topological diversities enable them to be tailored much more flexibly compared to an organic 2D materials toward desired properties for various application scenarios. To date, studies have been focusing on designing new crystalline 2D polymers and modulating linkage with different monomer combinations. Zeng et al.^[^ ^25^ ^]^ synthesized a 2D polymer by an irreversible strategy and adapted AFM nanoindentation to measure the elastic modulus and fracture strength of (12.7 ± 3.8) GPa and (488 ± 57) MPa, respectively. Hao et al.^[^ ^26^ ^]^ determined the elastic modulus of ≈25.9 GPa for COF_TTA‐DHTA_ applying the same method. Fang et al. investigated the elastic properties of two types of COFs with various thicknesses using AFM nanoindentation.^[^ ^27^ ^]^ However, the AFM method has been inappropriately served for evaluating the failure mechanisms of 2D polymers, because of three facts: first, since most of large area 2D crystalline polymers are polycrystalline system where the nanoindentation approach tends to overestimate the strength. The strength estimation will also vary as the indenter location changes along the grain boundaries.^[^ ^28^ ^]^ Second, although the rupture indication is able to be reflected by the force‐displacement curve, the complex strain distribution with large gradients around the tip implies that AFM measurements are more accurate in the elastic state, while the crack initiation and propagation are invisible either, which is vital for unveiling the failure mechanism. Last, the sophisticated calibration before indentation will strongly influence the final results. Therefore, it is trending and more precise to employ uniaxial tensile stress approach to identify more key properties in 2D polymers. Pantano et al.^[^ ^29^ ^]^ applied uniaxial tension on a 2D COF nanofilm to obtain more comprehensive information of mechanical response. The elastic modulus and the strength were measured as (37 ± 15) GPa and (188 ± 57) MPa, respectively. Fang. et al. visualized the fracture behavior of 2D COF in the scanning electron microscope (SEM), quantitatively determining the fracture toughness (0.55 ± 0.09 MPa√*m*) and confirming the existence of flaw‐insensitivity in 2D polymer system.^[^ ^30^ ^]^ However, probing structural change and fracture kinetics down to the molecular level remains challenging, and the underlying failure mechanism of crystalline 2D polymers still has to be explored, mainly due to the unsolved challenge of reserving the lattice structure from the sample preparation and transfer.

In this article, a nanoscale in situ mechanical study on 2D polyimine in the transmission electron microscope (TEM) is presented. A tensile force is applied to the 2D polyimine film and elastic response and fracture behavior are evaluated and imaged. The kinetics of fracture is described and material properties such as elastic modulus and critical stress intensity factor are determined. Particularly, an interesting phenomenon of transgranular fracture orientation is observed and deeply understood by density functional‐based tight binding (DFTB) simulation. Our findings offer solid knowledge from mechanical failure perspective for a general design strategy of 2D COFs for future applications.

## 2 Results and Discussion

### 2.1 2D Crystalline Membrane Synthesis

**Figure 1:**
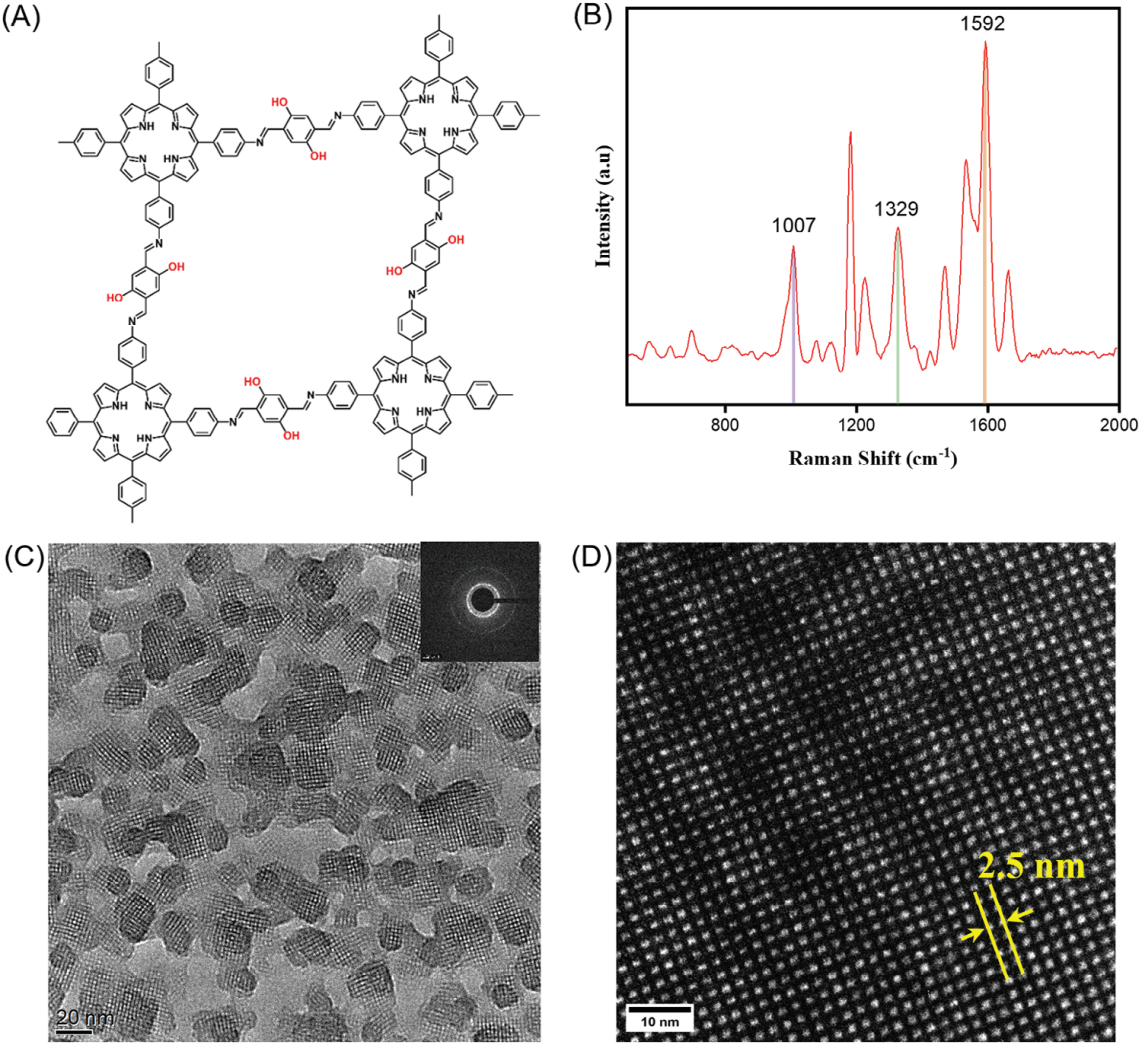
Structural information and characterization of 2D crystalline polyimine films. A) Chemical structure of 2D polyimine. B) Raman spectrum of a 2D polyimine film. C) TEM image of a 2D polyimine film, inset is the corresponding selected area electron diffraction pattern. D) HRTEM image of a single 2D polyimine crystal, with the lattice spacing of 2.5 nm.

The crystalline 2D polyimine membrane was synthesized at the water‐air interface applying a surfactant‐monolayer‐assisted interfacial synthesis method.^[^ ^31^, ^32^, ^33^ ^]^ A chloroform solution of sodium oleyl sulfate (SOS) was first dropped and spread on the water surface. Due to the quick evaporation of chloroform, the SOS molecules self‐assembled by the SO_4_^−^ head groups downward and formed a monolayer as a template for following polymerization. An aqueous solution of 5,10,15,20‐tetrakis (4‐aminophenyl)‐porphyrin (TAPP) protonated by triflic acid was subsequently injected into water at 50 °C. TAPP monomers were then guided and fixed underneath the SOS monolayer by hydrogen bonding and electrostatic interaction from SO_4_^−^ head groups. Finally, 2,5‐dihydroxyterephthalaldehyde (DhTPA) was added in the water phase. After its diffusion to the surface, the Schiff‐base condensation between amino and aldehyde groups was triggered, and the imine bond was formed between them. This interfacial reaction was continuously carried out at 50 °C for 5 days to generate a crystalline polyimine ultrathin film. The schematic illustration is depicted in Figure S1 (Supporting Information). The scalable synthetic strategy enables large area films with tunable thicknesses (Figure S2, Supporting Information). The designed chemical structure in **Figure** **1**A is confirmed by Raman spectroscopy, electron diffraction, and high‐resolution TEM, see Figure 1B–D. The synthesized film was characterized by Raman spectroscopy to identify the chemical structure of polyimine and to confirm the formation of the imine bond. As shown in Figure 1B, the peaks at 1007 cm^−1^ and 1329 cm^−1^ correspond to the ν (pyrrole breath) and ν (pyrrole quarter‐ring) in TAPP, respectively. The characteristic peak at 1592 cm^−1^ represents the stretching vibration of the ‐C = N‐ bond that proves the formation of the imine bond between TAPP and DhTPA monomers through the Schiff‐base condensation reaction.^[^ ^34^, ^35^ ^]^ TEM image and the inset corresponding diffraction pattern in Figure 1C show the high crystallinity of 2D polyimine with a domain size of ≈20 nm in diameter. High‐resolution (HR) TEM image in Figure 1D furtherly proves the structure with the lattice spacing of ≈2.5 nm along [100] / [010].

### 2.2 Sample Preparation

The most common technique for the sample preparation is focused ion beam (FIB),^[^ ^36^, ^37^, ^38^ ^]^ but based on our previous study, it will generate structural damages and edge spikes on 2D polymers, which are harmful for the acquisition of crystallographic information and quantification of intrinsic properties on the remaining tested sample. By evaluating the damage of a 2D polyimine membrane caused by focused Xe^+^ ions, it was found that FIB milling destroys the structure on the membrane and changes the edge morphologies (an amorphized milling trace and generation of spiky edges with affected areas in the dimension of hundreds of nanometers, see Figure S3, Supporting Information) even for a FIB line scan at low beam current. Since zeroing structural damage of the specimen is necessary for protecting the structure that is crucial to quantify the properties in 2D materials,^[^ ^35^ ^]^ the FIB method^[^ ^12^, ^14^, ^30^ ^]^ is not applicable for patterning 2D polyimine specimen. In contrast, patterning with a focused electron beam preserves the structure of the specimen, however, the speed for patterning is too low and carbon contamination is an unavoidable effect.^[^ ^39^ ^]^ Due to the above‐mentioned downsides of beam‐based sample preparation and to properly transfer the membrane from the beaker and fabricate a defined geometry, a beam‐free strategy was developed and applied in this study. First, 0.1 g polyvinylpyrrolidone (PVP) powder was dissolved in 10 mL H_2_O to prepare the PVP solution. A few drops of this solution were spin coated on a SiO_2_ / Si substrate at 2 000 rpm for 1 min. Next, the PVP‐coated substrate was used to carefully fish out 2D polyimine membrane from the beaker. To protect the polyimine specimen and to enhance the stiffness of the sample during the transfer process, a thin layer of poly methyl methacrylate (PMMA) was spin coated on the polyimine / PVP / substrate, with a spin rate of 3 000 rpm for 1 min. The assembly was kept at room temperature for 12 h. Subsequently, a sharp steel probe installed in a probe station of an optical microscope was employed to carve the assembly of PMMA / polyimine / PVP / substrate into ribbons, with lengths and widths of ≈100 µm and ≈20 µm, respectively. Then the carved assembly was immersed into the distilled water at a proper feeding angle (≈45°) with respect to the water surface. Because PVP is water soluble, a gap was formed to dissolve PVP. Water can easily penetrate to the interface from the gap due to the capillary force. Delamination and peeling the ribbons off from the substrate with a controlled rate results in a reduction of mechanical force needed. Next, a “Push‐to‐Pull (PTP)” device (Bruker)^[^ ^39^ ^]^ was treated by oxygen plasma for 10 mins to make the surface hydrophilic. The ribbon was manipulated, aligned, and driven toward the target gap on the device by utilizing the water. The patterning and transferring process have been depicted in Figure S4 (Supporting Information). After being annealed at 130 °C on a hot plate to ensure that the specimen firmly clamped through sample adhesion, the device was then soaked gently in hot acetone for 1 min to remove the PMMA layer, leaving behind the 2D polyimine film on the target position. The sufficiently large contact area (≈1.16 × 10^3^ µm^2^) between the tested film and PTP device with respect to the stretched area ensures the firm adhesion to avoid significant slippage.

### 2.3 In Situ TEM Tensile Test on 2D Polyimine Films

**Figure 2:**
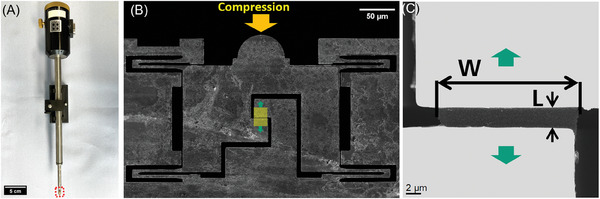
In situ TEM experimental setup and characterization of 2D polyimine specimen before test. A) An optical microscope image of PI95 TEM holder, the red dashed rectangle shows the position of PTP device. B) A SEM image of PTP device, the yellow arrow indicates the compression load direction, and green arrows show the tensile load exerted on the sample. C) A STEM dark field image shows the sample geometry before the tensile test.

To perform the in situ tensile test in TEM, a Bruker/Hysitron PI 95 in situ TEM Pico Indenter device (**Figure** **2**A) with a conductive diamond flat punch indenter controlled by a piezoelectric ceramic sensor was used to apply the load on the device where the compression load is converted into uniaxial tensile load on the freestanding specimen. The PTP device (see the SEM image of the device in Figure 2B) was then mounted in the in situ TEM holder and transferred inside a TEM (Carl Zeiss LIBRA 200 MC Cs TEM) to perform the test. Imaging during the in situ test was done at an acceleration voltage of 200 kV. The specimen was examined before testing to ensure that no significant flaws disturbed the tensile experiment. The PI95 in situ transducer was calibrated before each test by measuring applied force and displacement of indenter not in contact with the PTP device. Then, the in‐plane loading direction and the out‐of‐plane direction of the contacting area on the semi‐circle part were carefully aligned to ensure the indenter can stably apply the load. The stiffness of the PTP device was extracted from the recorded force‐displacement curve by conducting an additional loading‐unloading cycle after the breakage of the sample. The load‐displacement curve was recorded during the observations. After combining with the dimensions (length *L* and width *W* shown Figure 2C, which are measured in the scanning transmission electron microscope (STEM) images respectively taken before each test), the stress‐strain curve can be obtained for further analysis of mechanical properties, and elastic modulus was calculated from the linear part of the curve. Here, the stress was calculated by σ  = *F*/(*Wt*) , where *F* and *W* are the measured in‐plane force and initial width, respectively. The thickness *t* of suspended samples is ≈19 nm given by AFM measurements in Figure S2B (Supporting Information). The strain was calculated by ε  = Δ*L*/*L* , where Δ*L* is the displacement and *L* is the initial length of tested samples,^[^ ^14^ ^]^ and the strain in this work follows along the loading direction as ε_*xx*_. It is necessary to note that the behavior of 2D materials under tensile loading should be properly described by 2D in‐plane stress and strain, as well as the elastic modulus (which is 2D stiffness) with units of force/length. But for 2D multilayered materials, the tested specimen has a real “cross section” undergoing the tensile load considering the van der Waals (vdW) forces and π − π stacking. Normally these quantities are divided by summed interlayer spacing (thickness *t*) in order to obtain the corresponding 3D parameters for purposes of comparison to other materials.

### 2.4 In Situ Observation of Fracture

**Figure 3:**
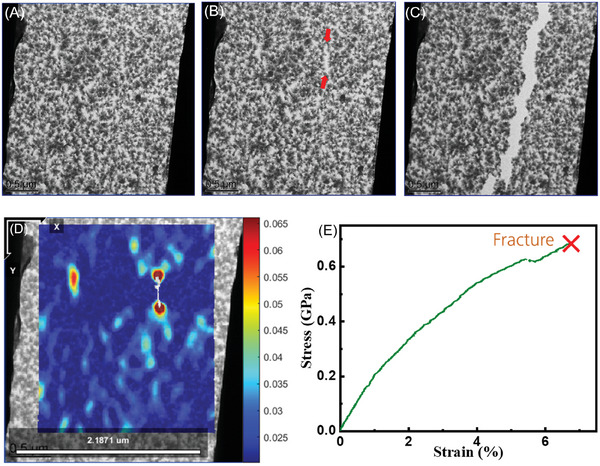
TEM images of fracture process. A) 0% strain. B) 6.9% strain before fracture. C) Fracture morphology on the 2D polyimine. D) DIC analysis result showing the stain concentration for crack initiation. E) One typical set of stress‐strain curve.

The in situ tensile test was performed in TEM after setting up and calibration (Video S1, Supporting Information). **Figure** **3**A,B shows the states of polyimine membrane before applying the load as 0% strain and the maximum strain before fracture, and we can see that there is a crack opening before fracturing, as indicated by red arrows. Figure 3C is the ultimate fracture across the sample. To analyze the localized strain distribution on the membrane before the fracture, digital image correlation (DIC) analysis^[^ ^40^ ^]^ was employed on Figure 3B. It is a robust and versatile technique to investigate the material deformation at different length scales to nanoscale by using image registration algorithms to track the relative displacements of pixel points between a reference image (pristine stage) and a series of images taken at deformed stage.^[^ ^41^, ^42^, ^43^, ^44^, ^45^ ^]^ The result is shown in Figure 3D. The observations above are consistent with the “weakest‐link” statistical model,^[^ ^9^ ^]^ i.e., the strength of polycrystalline 2D material is most possibly dictated by the most unstable defect under the impact of global load condition and localized strain field. The stress‐strain curve is obtained in Figure 3E, and the corresponding elastic modulus *E* and fracture strain are determined as ≈10.9 GPa and ≈6.9%, respectively. **Table** **1** lists the geometry, fracture strength, and elastic modulus of tested samples, where the average elastic modulus is (8.6  ±  2.5) GPa. The stress‐strain curve in Figure 3E does not show strictly linear behavior. In previous studies of 2D COF films under uniaxial tensile loading condition, the nonlinear behavior is also observed in one imine‐linked COF film,^[^ ^29^ ^]^ but it is linear in another two COF films.^[^ ^30^, ^46^ ^]^ Since the fracture strain is not high, we first exclude the nonlinear elasticity in the constitution equation (like in graphene). There could be three possible reasons: first, different chemical structures and synthetic methodologies by inducing the growth of COF films in different ways could be significantly impactive; second, the irreversible loss of crystallinity during the sample patterning and transferring process can also play a vital role,^[^ ^47^ ^]^ since the nonlinear behavior is only obtained with beam‐free approaches and non‐chemical reactivation, and in our work; third, it is similar with non‐linear behavior of carbon fibers and some composites^[^ ^48^, ^49^ ^]^ that the increasing tensile stress will lead to the crystallite reorientation and constrain the deformation of crystallites. The presence of sparse structure and defects in 2D polyimine film allows this to be happened, because unlike other nanocrystalline systems, nanocrystalline polymers synthesized by the interfacial strategy have a certain amount of amorphous regions surrounding crystallites as defects and make the film sparse. It is also accounted for the relatively low elastic modulus and large scattering error, because only 3% decrement of structural defects in COF membrane leads to 50% reduction of its elastic modulus.^[^ ^50^ ^]^ It is worth to note that only the amorphous area between grains with large variation in thickness (in our work) is likely to be the nuclei of crack initiation. The 2D polyimine undergoes a stable elastic deformation stage up to ≈5.5% until a stress drop on the curve, indicating a subcritical crack event takes place before the ultimate fracture at 6.9%. This implies that the fracture of 2D polyimine film is different from that in other 2D materials.

**Table 1:** Geometry and mechanical properties of tested 2D polyimine films.

Sample	Length (µm)	Width (µm)	Fracture strength (GPa)	Elastic modulus (GPa)
1	2.7	19.9	0.4	7.5
2	2.2	12.7	0.3	6.8
3	2.7	18.4	0.7	11.6
4	1.5	22.0	0.4	5.7
5	2.5	20.8	0.2	11.6
Average			0.4± 0.2	8.6± 2.5

### 2.5 Multiple Cracking Behavior

**Figure 4:**
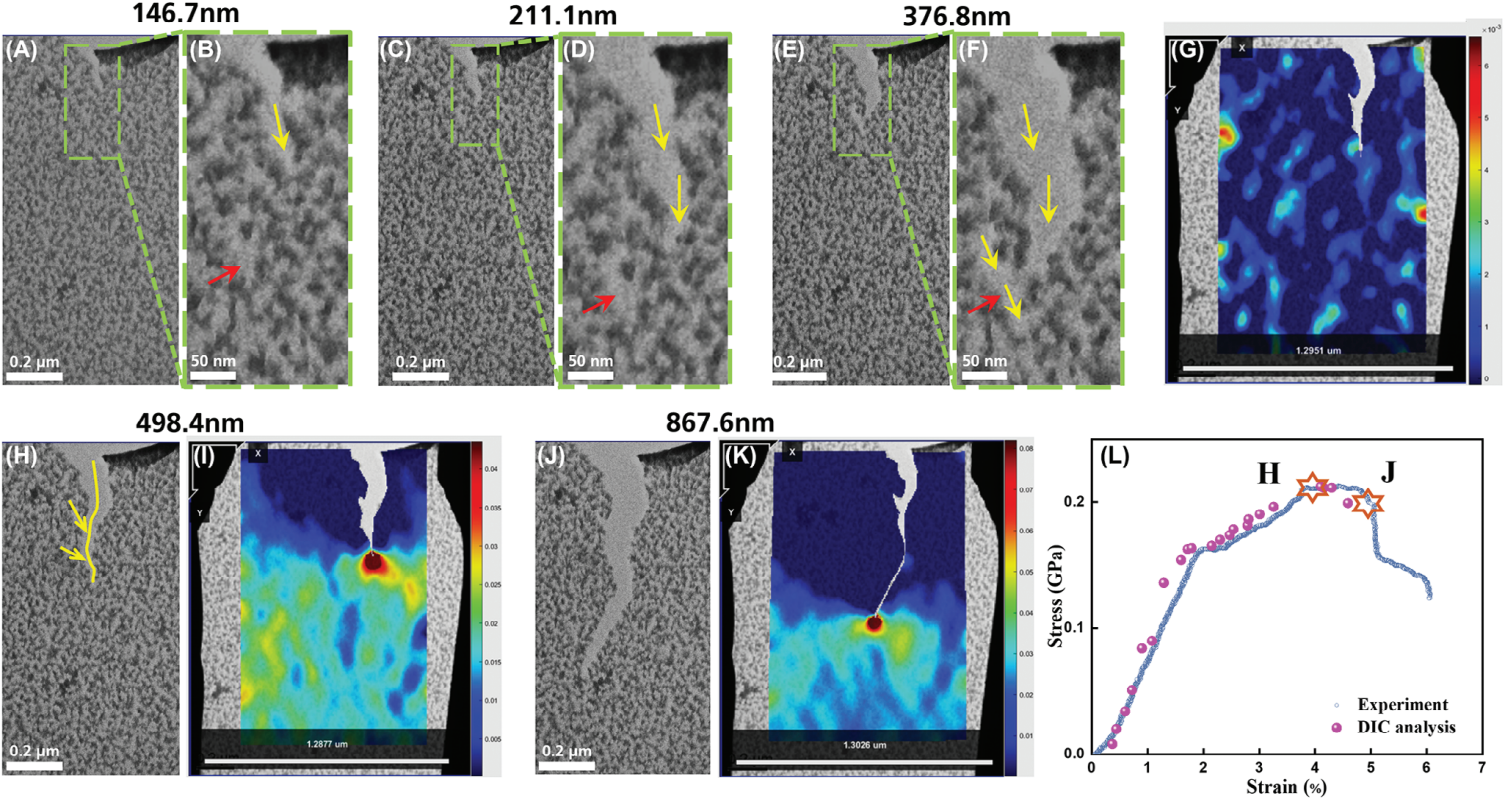
A–F) pre‐crack initiation and stable crack propagation with side crack development, crack lengths from 146.7 nm to 498.4 nm. G) the initial local strain distribution at the beginning of next stage. H,I) show the continuous crack growth from 498.4 nm to 867.6 nm until being arrested, J,K) map the corresponding strain evolution at crack tip. L) the corresponding stress‐strain curve, the magenta solid ball represents the local strain from DIC analysis.

To study the fracture mechanisms, multiple crack events have to be considered. **Figure** **4** shows the evolution of a stable crack propagation including several critical events. There is a crack at the edge with a length of 146.7 nm that was intentionally introduced as a pre‐crack in Figure 4A (Figure 4B shows the magnified image of pre‐crack in dashed rectangular area in 4A). As the stress continued to increase, this pre‐crack grew up to 211.1 nm (Figure 4C), and Figure 4D depicts the magnified structure in the vicinity of the crack tip opening simultaneously with the growth of pre‐crack, which is pointed by the red arrows. It can be clearly seen in Figure 4E,F that this opened structural defect developed large enough to merge into the path, which is supposed to be the pre‐crack passing through, but the remaining connection at the pre‐crack tip indicates that the propagation of pre‐crack was stopped before. This behavior suggests that the weak and sparse structures on the film would possibly evolve from existing flaws to the side cracks independently and join into the path as the main crack. This is contrary to the formation of branches in other 2D materials that are bifurcated from the main crack along the cracking direction and mainly caused by the asymmetric edge.^[^ ^17^ ^]^ After this stage, the pre‐crack was stable with the length of 498.4 nm. To quantitatively evaluate whether this cracking behavior can consume extra energy and toughen the film during the fracture process, fracture toughness needs to be obtained. Since experiments and simulations in previous studies consistently verified that the conventional Griffith theory of brittle fracture is applicable in 2D system,^[^ ^8^ ^]^ we then preliminarily calculate the critical stress intensity factor *K*_C_ taking account of the fracture strengths and semi‐lengths of the crack by applying Griffith theory expressed as:^[^ ^30^, ^51^ ^]^

(1) $$K_{C}=\;Y*\sigma_{f}*\sqrt{\pi *a_{0}}$$

where $Y=\left(1-0.025\alpha^{2}+0.06\alpha^{4}\right)*\sqrt{\sec\frac{\pi \alpha }{2}}$ is a geometry‐dependent empirical formula. As $\alpha \;=\frac{2a_{0}}{w}\;\ll 1$, so *Y* ≈ 1. σ_*f*_ is the critical stress, *a*_0_ is the semi‐length of pre‐crack. The *K*_C_ values are ≈0.15 MPa · *m* ^−2^ and 0.26 MPa · *m* ^−2^ for the critical cracking in 4C and 4E, respectively. This ≈73% increment of *K*_C_ indicates that more energy needs to be absorbed during this development of side crack for merging into the main crack. Furthermore, in order to analyze the local strain evolution during the crack growth in further stages, the initial state of strain is mapped in Figure 4G. Figure 4H,J shows that the side crack became the main one and grew up from 498.4 nm to 868.6 nm, and Figure 4I,K correspond to the critical strain distribution, respectively. The critical status of crack in 4H and 4J can be identified on the corresponding stress‐strain curve in Figure 4L. The local strain from a DIC analysis is plotted as well in magenta solid balls for comparison and for correction of the obtained engineering stress‐strain curve from the experiment. We can see that the stress increases from 0 up to 0.21 GPa when the strain is 4% at point H and there is a stress plateau from 4% to 5% strain from point H toward point J. It is obvious that the stress stays approximately constant when the crack tip is arrested in 4J and lasts for ≈1% strain, which is anomalous to other 2D materials which show brittle fracture. Note that the segment of the curve exceeding J point until ultimate fracture (from 5% to 6% strain) indicates that the similar arrestment of crack happened while it is impossible to calibrate the stress according to the corresponding crack lengths because of the limited field of view. DIC strain maps in Figure 4G,I,K indicate that the strain is more localized and concentrated at the crack tip when it is arrested at J compared with H. Since this is probably attributed to the difference grain structure in front of crack tip, and its toughness is highly determined by the grain boundaries in the vicinity of the propagating crack tip while the complex stacking and overlapping of monolayer in 2D polyimine makes the grain boundary more sophisticated. Therefore, the pathways of fracture among the individual layers are crucial yet seen and understood. It has been well investigated that 2D COF membrane tends to rupture along the grain boundaries after being mechanically damaged by a micromanipulator.^[^ ^52^ ^]^ This observation is in coincidence with our previous observation that intergranular fracture between polyimine crystal grains takes place without external load. However, it might be different in a dynamic situation, and the understanding of the fracture mechanism requires a more detailed imaging of the crack evolution.

**Figure 5:**
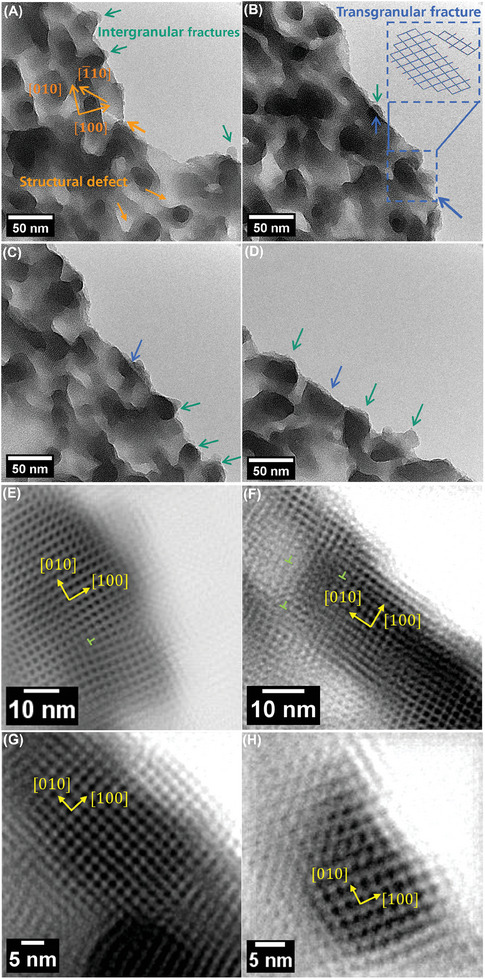
TEM images of several fracture types at the propagated crack edge, indicated by different arrows. A) a side crack and intergranular fracture paths near the propagated crack, structural defects are visible near the edge. B) dissimilar path among neighboring layers, and one typical transgranular fracture in blue dashed rectangle, inset is the corresponding sketch of crack path in the grain. C,D), the combination of several fracture types in a row. E,F), transgranular fractures with stepped topography and dislocations have preferential direction of [010] / [100] relative to the grain orientation. G,H), a smaller grain without dislocation emission.

To obtain more detailed structural information along the crack path, the crack edges were imaged at higher resolution after in situ testing as shown in **Figure** **5**. The crack edge is rough with a narrow bifurcation, which indicates a side crack along $\left[\bar{1}10\right]$ stopped propagating instead of cracking through the front grain (Figure 5A). Besides, structural defects are clearly visible near the propagated crack, and the round protrusions pointed by green arrows are distinct and intact grains and identified as intergranular fracture paths. According to the contrast inside and outside of the edge, the crack paths are dissimilar and independent among the layers, and this is attributed to the weak interlayer interaction of noncovalent π − π stacking, as well as the crystallographic independency of grain boundaries among layers. In Figure 5B, apart from the round path in one individual layer (green arrow), the straight path in its neighboring layer (blue arrow) implies that the crack path passes through the intact neighboring grain. The broken grain highlighted by the dashed blue rectangle strongly supports this assumption. The lower blue arrow points to the broken crystal that clearly shows the unfinished cracking path inside the crystal and inset is the corresponding schematic diagram of this process. Figure 5C,D shows the simultaneous presence of intergranular and transgranular fractures (indicated by green and blue arrows, respectively) on other sites of cracked edge as well. This observation experimentally proves that the transgranular fracture also exists in 2D polyimine under dynamic loading condition like polycrystalline graphene^[^ ^53^, ^54^ ^]^ although current research only determined intergranular fracture under static loading condition in 2D polyimine.^[^ ^52^ ^]^ Due to the reason that the toughness for intergranular cracking along suitably oriented grain boundaries is lower than that for macroscopic transgranular cracking in a polycrystalline aggregate,^[^ ^55^ ^]^ it allows dedicate grain boundary design to reduce intergranular cracking to increase the toughness for 2D polymers system. The transgranular cracking edges in Figure 5E,F exhibit the stepped topography, and the cores of edge dislocation emitted away from the cracked edge were captured. Since the high reversibility of condensation reaction of 2D polyimine endows the crystallites the defect‐correction mechanism, the dislocations are almost absent within each single grain on the pristine sample,^[^ ^56^ ^]^ and a few lone partial dislocations as the growth error are found only at antiphase grain boundaries in large grains.^[^ ^52^ ^]^ This strongly implies a disparate kinetics of dislocation emission during crack propagation. In monolayers TMDs, it has been observed that the stress at the crack tip changed the maximum direction midway due to far field complex grain boundaries, full dislocations or partial dislocations could be emitted from the crack front and blunt the crack tips.^[^ ^57^, ^58^ ^]^ This partially explains why not all the transgranular cracking path is accompanied with dislocation emission, particularly in relatively small grains where the stress at crack tip can hardly turn oblique as shown in Figure 5G,H. Another possible reason is the edge stacking dislocation. It is generated in bilayer graphene by stretching of one of layers and leading to a change of stacking order,^[^ ^59^, ^60^ ^]^ which is likely to happen in multilayered 2D polyimine while it still needs further explored. More interestingly, all the trangranular cracking path preferentially follows the cleavage orientation of [010] / [100]. To elaborate this mechanism and how it impacts the transgranular cracking behavior, Density Functional based Tight binding (DFTB) calculations were employed.

### 2.6 Fracture Mechanism

**Figure 6:**
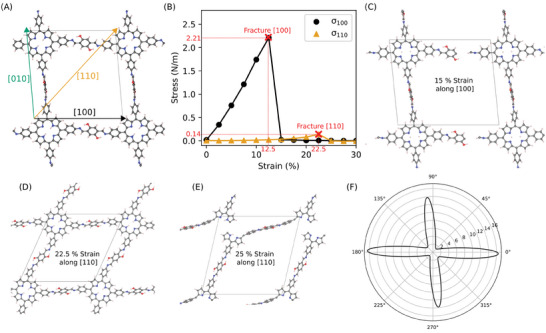
A) 2D polyimine with depiction of the [100], [110] and [010] direction. B) Strain‐Stress curves for the [100] and [110] strain directions. C) Depiction of the fracture of the [100] strain. D) Strained pristine structure along [110]. E) Fractured structure along [110]. F) Spatial dependence of the 2D elastic modulus based on ±0.4% strain calculated from the 2D stiffness tensor (Table S1, Supporting Information) with the ELATE code.^[^ ^62^ ^]^

The DFTB simulation results are shown in **Figure** **6**. Applying a tensile strain on a polyimine unit along different crystallographic orientations (Figure 6A), we can see that the [100] direction has a linear response from 0% to the fracture point 12.5% with a corresponding 2D fracture strength of 2.21 N m⁻^1^ (Figure 6B). The fracture originates from the breakage between C_HQ_ ‐C_I_ bond between the hydroquinone unit and imine bond because of the lowest dissociation energy compared to other bonds as shown in Figure 6C and Figure S5 (Supporting Information). In contrast, straining along [110] has a very low stress‐strain response and the first partial fracture occurs at a much higher strain of 22.5% (Figure 6E). From the calculated 2D stiffness tensor (Tables S1–S4, Supporting Information) the directional dependence of the elastic modulus is obtained and shown in Figure 6F. Here, the highly anisotropic behavior of the 2D polyimine is apparent with a maximum 2D modulus of 16.48 N m⁻^1^ along [100] and a minimum 2D modulus of 1.18 N m⁻^1^ along [110], i.e., approximately one order of magnitude difference. Additionally, by visualizing the extended structure at 22.5% strain, we can see in Figure 6D that the unit cells are significantly stretched and distorted while there is no structural distortion when they are strained along [100] / [010]. These different responses can be interpreted that stress direction selectively contributes to the transgranular fracture. Although theoretically the required dissociation in direction of [110] is the same with that in [010] / [100] by breaking the C_HQ_ ‐C_I_ bond, the structural flexibility of [110] induces the deformation of polyimine structural pore to highly dissipate the elastic energy and avoid any stress concentration on the structure. Therefore, the C_HQ_ ‐C_I_ bonds are well protected from the breakage. However, this dissipation does not happen along [100] / [010] due to its different topological feature. So, after initiating the C_HQ_ ‐C_I_ bond breakage and forming the crack, it will follow [100] / [010] direction and grow. It is obvious that the global stress response on 2D polyimine film is primarily contributed by [100] / [010], and the ideal case of preventing the fracture in polyimine film is that all crystallites are [110] oriented with respect to the external loading direction. More importantly, it is not difficult to correlate this with different fracture types observed above. When the crack moves toward a grain with respect to its [110] direction, low stress response from [110] will lead to a structure deformation which suppresses the concentration on the grain, and thus the crack tip deflects and continues the propagation intergranularly. In this case, strengthening the grain boundaries, e.g., by introducing interwoven structure as the uniform amorphous connection among grains, serves a valid strategy to enhance the 2D polymers.^[^ ^61^ ^]^ While in the case with respect to [100] / [010], the strain concentration on the front grain cannot be distracted and consequently introduces the transgranular fracture, where the grain boundary engineering may not work. This difference in fracture path will be translated to the macroscopic properties and have implications for the design of 2D polymers that we could well steer their crack behavior to facilitate more specific applications.

### 2.7 Comparison of Computational and Experimental Results

Based on the computational evaluation of the interlayer spacing in the bulk layered 2D polyimine of 0.3116 nm (Figure S6, Supporting Information), we can estimate that there are ≈61 layers in ≈19 nm thick 2D polyimine film (with a certain deviation due to differences of theory and experiment). We can also approximate from the 2D modulus of monolayer polyimine film a 3D elastic modulus that we can then compare with the experimental results as shown in **Table** **2**. It is worth noting that this approximation works reasonably well because of weak coupling between the organic interlayered materials, in our case it is π‐π interlayer stacking.

**Table 2:** Summary of the computational elastic properties, including the 2D Voigt, Reuss and Hill averages.^[^ ^63^ ^]^

Type	Direction	Fracture Strain	2D fracture strength (N/m)	2D elastic Modulus (N/m)	Fracture Strength (GPa)	Elastic Modulus (GPa)
Simulation (Pristine structure)	100	12.5%	2.54	16.48	8.16	52.88
	110	22.5%	0.14	1.18	0.46	3.78
	Voigt average	‐	‐	9.36	‐	30.04
	Reuss average	‐	‐	2.59	‐	8.32
	Hill average	‐	‐	6.06	‐	19.46
Experiment		(6.5± 2.4) %	‐	‐	0.4± 0.2	8.6± 2.5

Table 2 shows the comparison of elastic modulus, fracture strain and strength along different orientations of pristine films between experimental and computational results. It can be seen that the fracture strain in the experiment is approximately half of the simulation values along [100] / [010] while the fracture strength and elastic modulus are ten times lower than the computational value. This gives us a rough upper boundary on what elastic modulus could be maximally measured in the experiment. The reduced experimental modulus can be interpreted by considering on the one hand the density of inhomogeneities (defects, variation of thickness, etc.) and on the other hand, the alignments of the grains which are not along [100] / [010]. According to the simulation (see Figure S7 and Table S1, Supporting Information), the effect of introducing defects via removal of core or linker molecules was studied computationally and compared, which indicates that defects have little influence on the fracture strain but can have a significant influence on the fracture strength and the general elastic properties.

To have a better approximation regarding the influence of the polycrystallinity of the sample, the 2D Voigt, Reuss and Hill averages were calculated. Here, the experimental Young's modulus is close to the computational Reuss average which is a lower boundary of the averaged modulus, and hence, is in excellent agreement.

Although in reality three modes of stress along three directions exist from surroundings because of the randomly oriented crystallites in the polycrystalline film, it can still inspire us about more rational design on chemical structure of 2D polymers to strengthen their mechanical reliability by avoiding or delaying the detrimental failure along certain orientations, e.g., more complex topology design to disperse the stress concentration on the most fragile directions ([100] / [010] in 2D polyimine), direction synthesis to enable structural deformation for all directions, and film morphology design, etc. More importantly, this conformational variation significantly enables unconventional behavior in gas sorption and separation benefiting from the internal pore variation,^[^ ^64^ ^]^ and up to 40% framework expansion triggered by solvent absorption has been achieved in a dynamic and pore‐deformable COF,^[^ ^65^ ^]^ which can also be possibly tuned mechanically as we proved. Note that the similar structural behavior was predicted in the hexagonal nano pored COFs to impact out‐of‐plane resistant behavior and adhesion modulation.^[^ ^66^ ^]^ But to our best knowledge, it is the first time to visualize this effect on the cracking path and fracture behavior of 2D crystalline polymers. By obtaining the good consistency between DFTB simulation results and in situ observation, we comprehensively unveil the fracture behavior of 2D polyimine film and the impact of chemical structure on failure mechanism of 2D polymer.

## 3 Conclusion

To summarize, a robust patterning and transferring technique on 2D crystalline polyimine films has been exploited and enables the successful in situ in‐plane tensile tests within a TEM and the fracture behavior observation. The elastic modulus of polycrystalline 2D films synthesized by interfacial strategy was investigated to be (8.6 ± 2.5) GPa, and the fracture strain and stress are (6.5 ± 2.4) % and (0.4 ± 0.2) GPa, respectively. Multiple cracking events were consecutively observed to uncover the step‐by‐step cracking behavior at crack tip. The side crack initiation in the vicinity of the crack tip can result in substituting the existing main crack, which consequently contributes an increase of the critical stress intensity factor by ≈73%. The strain evolution during the fracture process was able to be mapped and visualized by DIC analysis. The intergranular fracture and dissimilar pathways among the layers were found at the crack edge, and more interestingly, our study unprecedentedly reveals the occurrence of distinct transgranular fractures during dynamic loading, and the preferential cracking direction in transgranular fracture is along the [100] / [010]. This arises from the topological flexibility of [110] direction and results in an exceptionally low stress response and higher fracture strain compared to [100] / [010], which was well substantiated by DFTB simulation.

Our findings not only deepen the understanding of the intrinsic mechanical properties and of the fracture behavior of 2D polyimine thin film, but also established how the chemical structure of 2D crystalline polymer contributes to their mechanical failure. It provides valuable insights for the general design of 2D polymers and illuminating new synthesis strategies in terms of mechanical reliability.

## Conflict of Interest

The authors declare no conflict of interest.

## Author Contributions

B.Z. and Z.L. designed and conducted the in situ experiments. X.L., W.L., R.D., and X.F provided and synthesized 2D polyimine films. J.L performed Raman spectroscopy and AFM measurements. B.Z patterned the 2D films and transferred the specimens on the PTP device and performed the data processing and DIC analysis. D.B., A.D., and G.C. conducted the DFTB calculations. The manuscript was prepared and revised by B.Z., Z.L., A.C., B.J., and E.Z. with inputs from all co‐authors.

## Supporting information



Supporting Information

Supporting Information

Supporting Information Video 1

Supporting Information Video 2

Supporting Information Video 3

Supporting Information Video 4

Supporting Information Video 5

Supporting Information Video 6

Supporting Information Video 7

## Data Availability

The data that support the findings of this study are available from the corresponding author upon reasonable request.
