# Supplementary material for: Fracture Behavior of a 2D Imine‐Based Polymer
Source: Adv Sci (Weinh). 2024 Sep 12;11(42):2407017. doi: 10.1002/advs.202407017 (PMC11558127; doi:10.1002/advs.202407017)
Supplement: Supplementary file 1 — Supporting Information [file ADVS-11-2407017-s001.docx]

Supporting Information

Fracture behavior of a two-dimensional imine-based polymer

*Bowen Zhang, Xiaohui Liu, David Bodesheim, Wei Li, André Clausner, Jinxin Liu, Birgit Jost, Arezoo Dianat, Renhao Dong, Xinliang Feng,* *Gianaurelio Cuniberti, Zhongquan Liao*, Ehrenfried Zschech*

**This document includes:**

- Experimental details
- Computational details
- Figure. S1 to S7
- Table S1 to S5
- References

# Experimental details


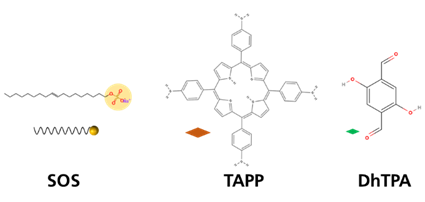


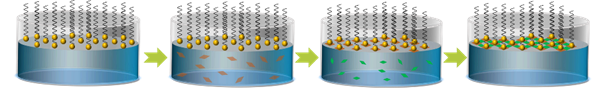


**Figure S1**. Schematic illustrations for the synthesis of 2S polyimine film through SMAIS method


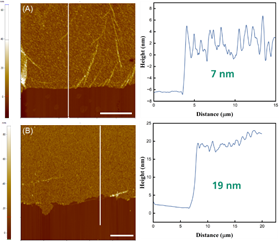

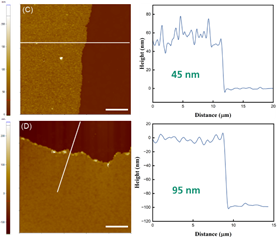


**Figure S2**. Atomic force microscopy (AFM) images (left) of 2D polyimine films with various thicknesses. The corresponding height profiles along the white lines in AFM images are depicted on the right. (**A)** 7nm, (**B)** 19nm, (**C)** 45 nm and (**D)** 95 nm. Scale bar: 4 µm.


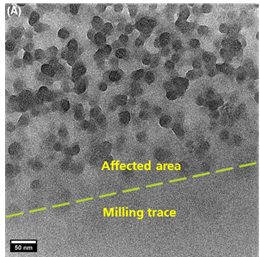

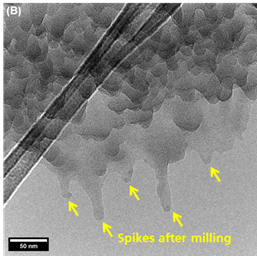


**Figure S3**. TEM images of the 2D polyimine fim patterned by FIB. **(A)** An amorphized trace milled at 1 pA and the affected area with structure damage on the crystallites. **(B)** An spiky edge milled at 10 pA, where the spikes are clearly visible.


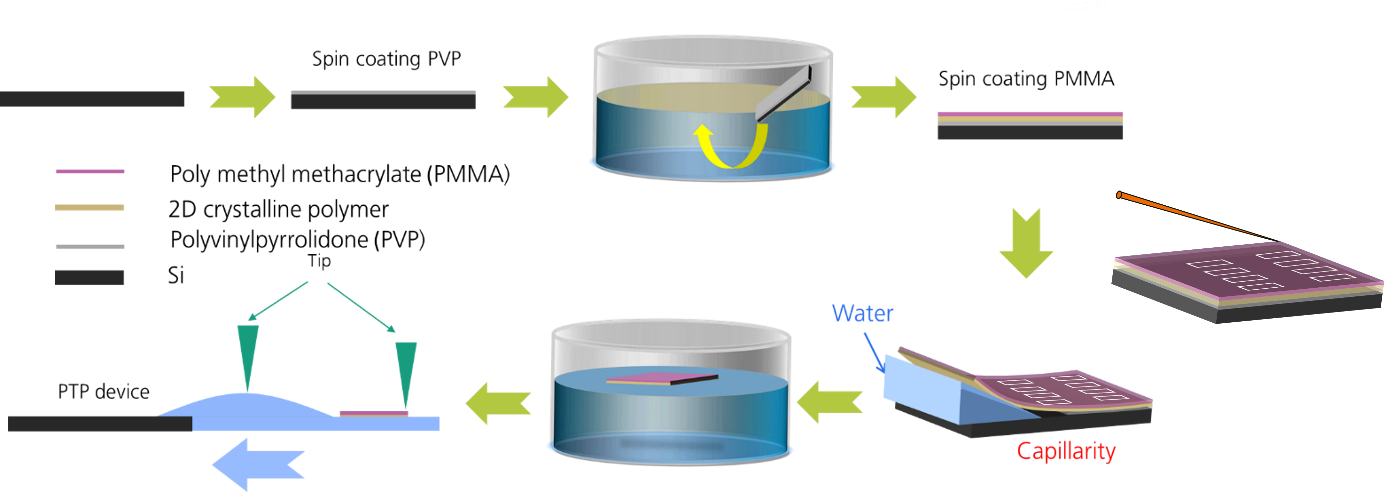


**Figure S4.** Mechanically patterning and transferring approach in a probe station.

# Videos

Videos of *in-situ* tests are available from the authors.

# Computational Details

All density functional based tight binding (DFTB) calculations were performed with the package DFTB+ ^[1]^. As Slater-Koster parametrization the matsci-0-3 parameters were used ^[2, 3]^ as well as a DFTD4 dispersion correction with the following parameters: s6=1.0, s8=3.3157614, s9=1.0, a1=0.4826330, a2=5.3811976 ^[^[^4^](https://doi.org/10.1063/1.5090222)^]^. Due to the system size, only one k-point was chosen, except for the bulk-system where along the stacking direction 5 k-points were used. The relaxation was performed with the atomic simulation environment (ASE) using the SciPyFminBFGS algorithm with a convergence threshold of a maximum force on all individual atoms of 0.01 eV/Å. For better convergence of the SCC cycles, an elevated temperature of 100 K for the Fermi Filling was used. For the [100] and [010] straining, the cell was rotated so that the respective directions are aligned along x. Then the cell was strained along x while the y-direction was relaxed. This way, all stress components, except for σ­_xy_, are being minimized. To be consistent with the experimental measurements, the stress was normed by the initial width, i.e., the engineering stress.

For the calculation of the full stiffness tensor, the central difference approximation to the derivative of the stress tensor is used. Here, the 2D polymer is strained along different directions with ±0.4% strain, and the stress tensor is calculated after the relaxation of the atomic positions. From this, the Voigt stiffness tensor is constructed from which other elastic properties can be derived. This procedure is based on the implementation in the Computational 2D Materials Database ^[5, 6]^.

For the bond-breaking analysis, a fragment of the 2D polymer was considered consisting of two connected core molecules with a linking molecule. The interatomic distance between the two bonding atoms was increased in 0.1 Å increments and fixed via the ASE FixBondLength constraint. At each step, the geometry was relaxed with the positions of the two bonding atoms being fixed. In order to prevent the collapse of the fragment, the two terminal C atoms were fixed regarding their interatomic distance. As shown in Figure S4, the bond dissociation energies are depicted for different bond-types in the system. The C_HQ_-C_I_ single bond has the lowest dissociation energy. This is also the bond that cleavages in the straining simulations of the 2D polymer. The C-N bonds are stronger and are less likely to break due to mechanical stress. This shows that the crack propagation mechanism is most likely due to the bond-breaking of the central C-C single bond.


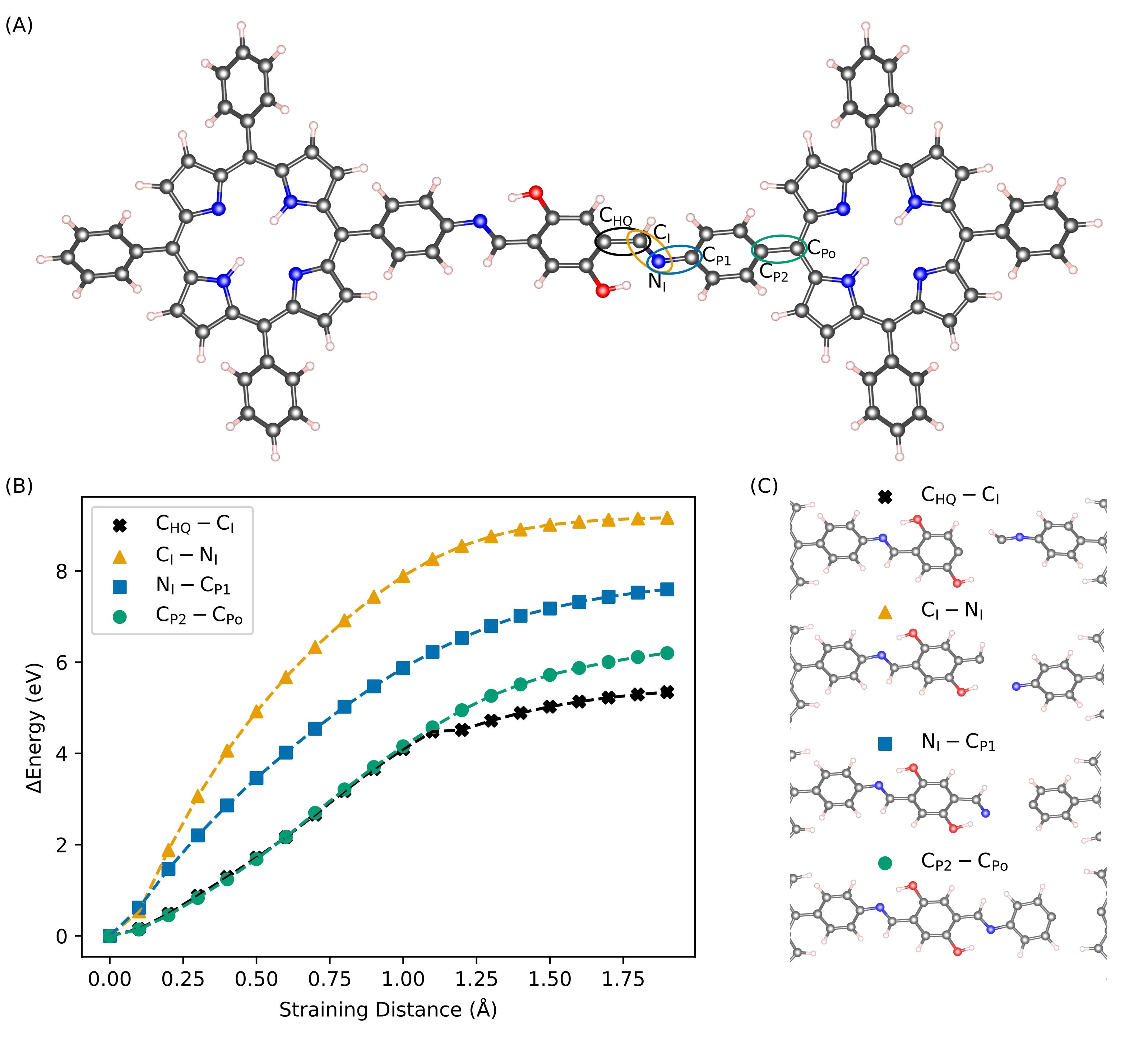


**Figure S5.** **(A)** Structure of the fragment of the 2D Polymer used for the bond-breaking analysis. The atoms are labelled as following: C_HQ_ is the Carbon of the Hydroquinone unit, C_I_ and N_I_ are the Carbon and Nitrogen of the imine bond, C_P1_ and C_P2_ are Carbon atoms of the phenyl ring, and C_Po_ is the Carbon of the Porphyrine unit. **(B)** Dissociation curves of the different bond-cleavages. **(C)** Depiction of the broken bonds.


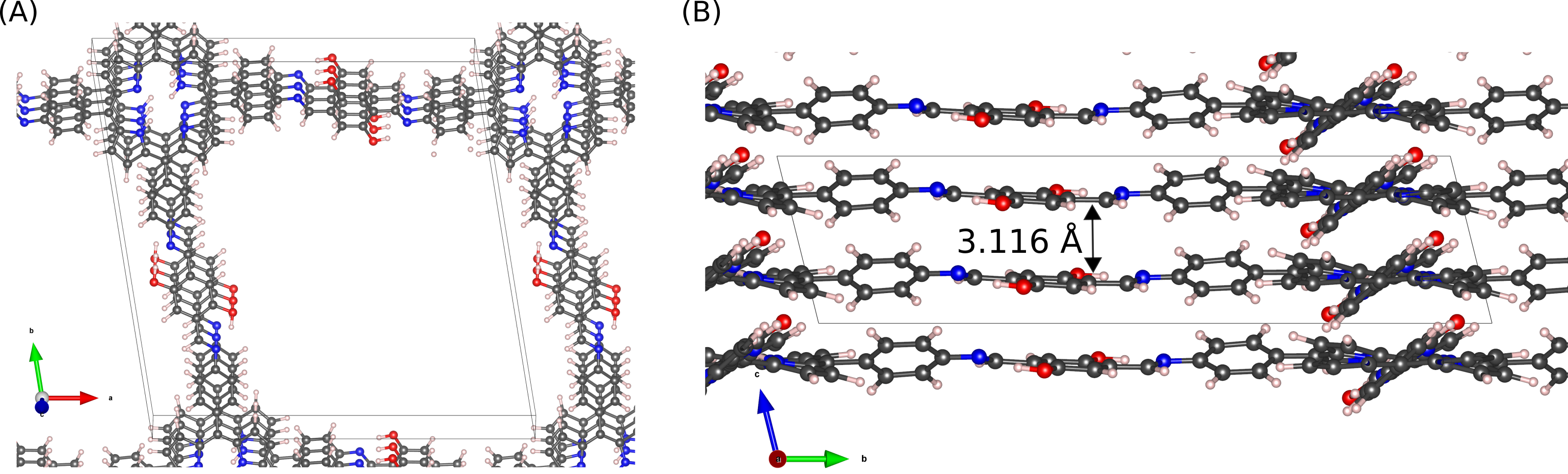


**Figure S6:** **(A)** Top view of calculated bulk-structure of 2D polyimine with cell-parameter a=25.479 Å, b=25.426 Å, c=6.432 Å, α=103.8°, β=90.5°, γ=99.2°. **(B)** Side view of calculated bulk-structure of 2D polyimine with an interlayer-distance of 3.116 Å.

Defective unit cells were also strained in the simulation to evaluate the defect impact on the mechanical behavior of 2D polyimine in comparison of the pristine unit cell. To probe the influences of defects in this system, two types of defects were created out of a 2x2 supercell, as shown in Figure S6A and S6B. We name Type I as linker-defect if a DhTPA molecule is missing and Type II as core-defect if a TAPP molecule is missing. As expected, the stress response along [010] is virtually unchanged in the case of the linker-defect, as the number of linkers responsible for resisting the strain stays constant. It is halved along [100] as here half of the linkers are available. The fracture point, however, stays the same. The [110] stress response shows a reduction as well. The core-defect shows a halved strain-stress response along [100] and [010] due to the halved number of connection points along each of the directions. Along [110] stress response is also strongly reduced, but due to the interaction between the dangling linker molecules during the shearing, no obvious trend can be determined


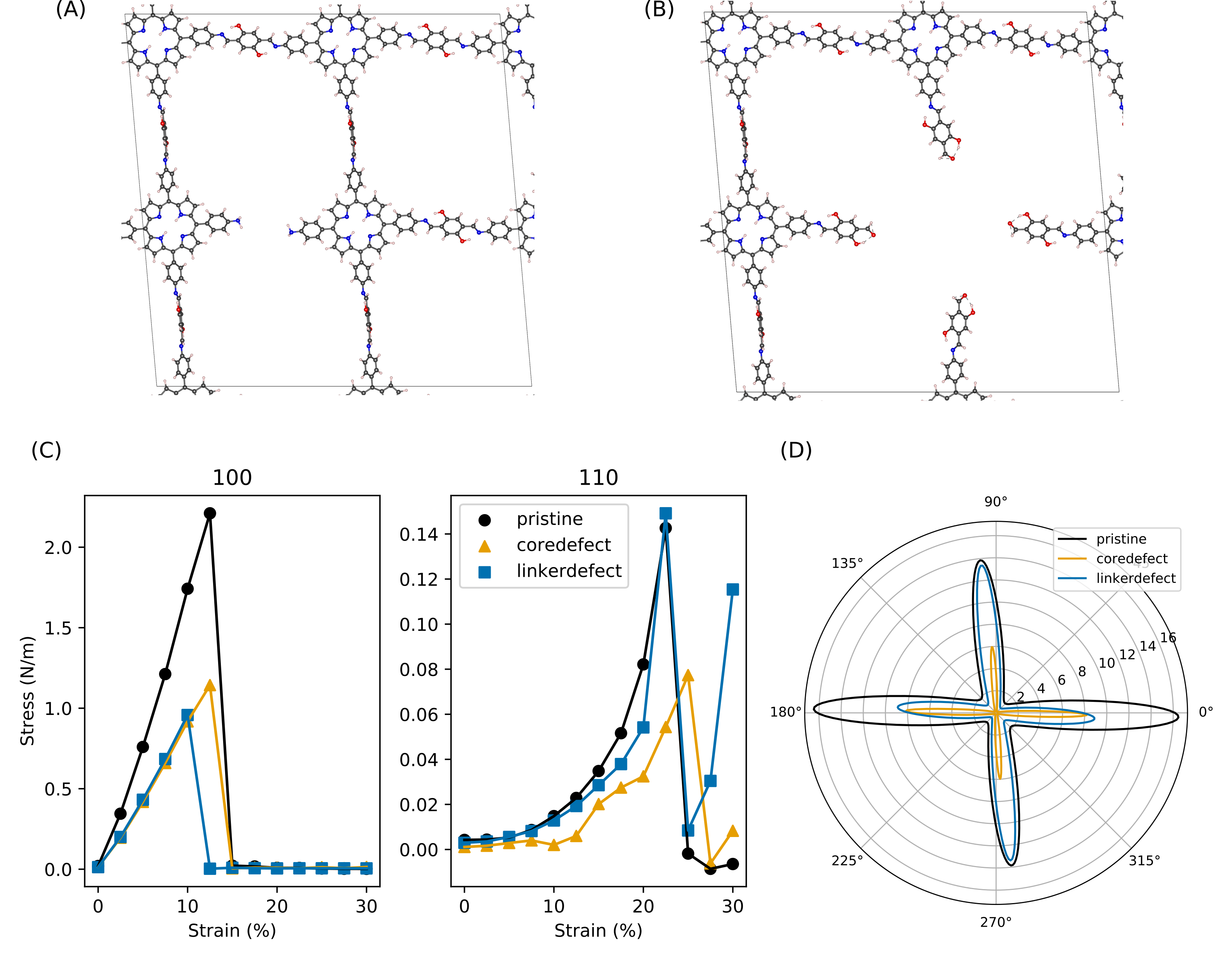


**Figure S7:** **(A)** Structure of the linker-defective system. **(B)** Structure of the core-defective system. **(C)** Strain-Stress responses along different directions for the pristine, linker-defective and core-defective system. **(D)** Spatial dependence of the elastic modulus based on ±0.4% strain calculated from the 2D stiffness tensor**.**

**Table S1.** Summary the elastic properties of the pristine, linker-defective and core-defective structures. The values of the 2D elastic modulus were obtained from the calculated stiffness tensors (Table S2, S3, S4).

| **Type** | **Direction** | **Fracture  Strain** | **Fracture Strength [N/m]** | **Elastic Modulus [N/m]** | **Fracture  Strength [GPa]** | **Elastic  Modulus [GPa]** |
| --- | --- | --- | --- | --- | --- | --- |
| Pristine | 100 | 0.125 | 2.54 | 16.48 | 8.16 | 52.88 |
|  | 010 |  |  | 13.84 |  | 44.41 |
|  | 110 | 0.225 | 0.14 | 1.18 | 0.46 | 3.78 |
|  | Voigt |  |  | 9.36 |  | 30.04 |
|  | Reuss |  |  | 2.59 |  | 8.31 |
|  | Hill |  |  | 6.06 |  | 19.45 |
| linker defect | 100 | 0.100 | 0.96 | 8.89 | 3.07 | 28.54 |
|  | 010 |  |  | 13.38 |  | 42.95 |
|  | 110 | 0.225 | 0.15 | 0.58 | 0.48 | 1.88 |
|  | Voigt |  |  | 7.22 |  | 23.16 |
|  | Reuss |  |  | 1.37 |  | 4.40 |
|  | Hill |  |  | 4.45 |  | 14.29 |
| core defect | 100 | 0.125 | 1.14 | 8.19 | 3.67 | 26.27 |
|  | 010 |  |  | 5.95 |  | 19.10 |
|  | 110 | 0.250 | 0.08 | 0.11 | 0.25 | 0.35 |
|  | Voigt |  |  | 4.60 |  | 14.76 |
|  | Reuss |  |  | 0.25 |  | 0.79 |
|  | Hill |  |  | 2.64 |  | 8.47 |

**Table S2.** Calculated Voigt 2D Stiffness Tensor (N/m) for the pristine system

| 23.74 | 11.90 | -1.60 |
| --- | --- | --- |
| 11.90 | 19.42 | -2.05 |
| -1.60 | -2.05 | 0.60 |

**Table S3.** Calculated Voigt 2D Stiffness Tensor (N/m) for the linker-defective system

| 9.38 | 2.74 | -0.77 |
| --- | --- | --- |
| 2.74 | 13.87 | -1.45 |
| -0.77 | -1.45 | 0.37 |

**Table S4.** Calculated Voigt 2D Stiffness Tensor (N/m) for the core-defective system

| 8.28 | 0.78 | -0.23 |
| --- | --- | --- |
| 0.78 | 6.00 | -0.38 |
| -0.23 | -0.38 | 0.06 |

**Reference**

[1] B. Hourahine,B. Aradi, V. Blum, F. Bonafé, A. Buccheri, C. Camacho, C. Cevallos,

M. Y. Deshaye, T. Dumitrică, A. Dominguez, S. Ehlert, M. Elstner, T. van der Heide, J. Hermann, S. Irle, J. J. Kranz, C. Köhler, T. Kowalczyk, T. Kubař, I. S. Lee, V. Lutsker, R. J. Maurer, S. K. Min, I. Mitchell, C. Negre, T. A. Niehaus, A. M. N. Niklasson, A. J. Page, A. Pecchia, G. Penazzi, M. P. Persson, J. Řezáč, C. G. Sánchez, M. Sternberg, M. Stöhr, F. Stuckenberg, A. Tkatchenko, V. W.-z. Yu, T. Frauenheim, *J. Chem. Phys.*, **2020,** 152, 124101.

[2] S. Gemming, A. N. Enyashin, J. Frenzel, and G. Seifert, *Int. J. Mat. Res.*, **2010**, 101, 758–764.

[3] B. Lukose, A. Kuc, J. Frenzel, T. Heine, *Beilstein J. Nanotechnol*., **2010**, 1, 60–70.

[4] E. Caldeweyher, S. Ehlert, A. Hansen, H. Neugebauer, S. Spicher, C. Bannwarth, S. Grimme, *J. Chem. Phys.,* **2019**, 150, 154122.

[5] S. Haastrup, M. Strange1, M.Pandey, T. Deilmann, P. S. Schmidt, N. F. Hinsche, M. N Gjerding, D. Torelli, P. M. Larsen, A. C. Riis-Jensen, J. Gath, K. W. Jacobsen, J. J. Mortensen, T. Olsen, K. S. Thygesen, *2D Mater*., **2018**, 5, 042002.

[6] M. N. Gjerding, A. Taghizadeh, A. Rasmussen, S. Ali, F. Bertoldo, T. Deilmann, N. R. Knøsgaard, M. Kruse, A. H. Larsen, S. Manti, T. G. Pedersen, U. Petralanda, T. Skovhus, M. K. Svendsen, J. J. Mortensen, T. Olsen, K. S. Thygesen, *2D Mater*., **2021**, 8, 044002.
